# Health care seeking behaviour for visual dysfunction among motor vehicle drivers in Osun State, Southwest Nigeria

**DOI:** 10.11604/pamj.2019.32.17.16127

**Published:** 2019-01-11

**Authors:** Adefisoye Oluwaseun Adewole, Olufemi Ajumobi, Saheed Gidado

**Affiliations:** 1 African Field Epidemiology Network, Asokoro, Abuja, Nigeria

**Keywords:** visual dysfunction, health care seeking behavior, government drivers, commercial drivers, Southwest Nigeria

## Abstract

**Introduction:**

Visual impairment is recognized as a public health problem worldwide. People and generally drivers do not often go for routine medical or eye examination based on varied reasons. This study assessed health care seeking behaviour for visual dysfunction among motor vehicle drivers in Osun state, Southwest Nigeria.

**Methods:**

This was a comparative cross-sectional study among 120 male commercial and 120 government drivers, selected using multi-stage sampling technique in Osogbo, Osun State. Data on knowledge, attitude and health seeking behavior of drivers for visual dysfunction and barriers for not seeking medical treatment was collected using a pre-tested semi-structured questionnaire. Questions on awareness about visual functions and attitude of the respondents concerning visual problems were scored. Two sample independent t-test was used to elicit association between mean age/knowledge of government and commercial drivers and health seeking behavior for visual dysfunction.

**Results:**

The mean age of government and commercial drivers was 44.53years ± 8.51 and 38.52years ± 8.60 respectively. The mean knowledge (p<0.001) and attitude (p=0.001) differences of government and commercial drivers were associated with health seeking behavior for visual dysfunction. Of the 120 government drivers, 24 (20.0%) were aware of their current visual problems. Of the 24, government drivers, 10 (47.6%) visited the hospital for treatment. Busy workplace schedule (n = 5, 20.8%) and lack of awareness of visual defects ((n= 3, 12.5%) by commercial drivers were identified barriers for not seeking medical treatment for visual dysfunction.

**Conclusion:**

Knowledge and attitude towards visual dysfunction were higher among the government drivers compared to commercial counterparts. Government drivers had better health seeking behavior for visual dysfunction as compared to their commercial counterparts. We recommended routine eye medical check-up for early detection of visual dysfunction in motor vehicle drivers.

## Introduction

Visual impairment and blindness due to ocular diseases is recognized as a significant public health problem worldwide with devastating effect on the quality of life of individuals [1, 2]. According to World Health Organization, 285 million people are visually impaired globally, with 90% of this population living in developing countries [3]. Refractive error, cataract and glaucoma are increasingly being recognized worldwide as a significant cause of avoidable visual disability, as evidenced by their inclusion in the priority areas of the global initiative “VISION 2020”: The Right to Sight” to eliminate avoidable blindness [4]. Good vision is a fundamental component of safe driving, being one of the most important sensory factors for this activity, accounting for about 95% of all sensory requirements [5]. Drivers with good vision have an advantage over those with poor vision. Refractive error is a commonly reported ocular morbidity among drivers [6-8]. In Africa and generally in Nigeria, drivers do not often go for routine medical or eye examination. The vast majority attend hospitals when the condition becomes worse and after trying self-medications without success [9]. The problem of low uptake of eye care services in developing countries has been given lower priority compared to the need for resource provision. Evidence shows that even when eye care services are available, they are under utilised by potential beneficiaries [10-12]. Studies have accessed visual functions of commercial and those in public institutions respectively in relation to road traffic accidents, prevalence of visual dysfunction and barriers to utilization of eye care services [6-8, 13-15], in Nigeria however there is paucity of information regarding comparison of health seeking behaviours of commercial and government drivers in relation to their visual dysfunction. This study assessed knowledge, attitude and health care seeking behaviour for visual dysfunction among motor vehicle drivers in Osun state, Southwest Nigeria.

## Methods

**Study area:** we conducted a comparative cross-sectional descriptive study among intra-city commercial and state government drivers in Osogbo, Osun State from May to July 20^14^. Osogbo is the capital of Osun state, a state situated in the southwest Nigeria with a population figure of 3,416,959 [16]. Osogbo metropolis has three local government areas namely Osogbo, Olorunda and Egbedoore. The major means of intra-city transportation in Osogbo metropolis is the commercial intra-city mini-buses (commonly called “Korope”). The commercial intra-city mini-buses operating within Osogbo metropolis are under the jurisdiction of these local governments and are registered under three supervisory bodies; National Union of Road Transport Workers (NURTW), Road Transport Employers Association of Nigeria (RTEAN) and Cooperative Union of Road Transport Workers with each having executives headed by a chairman that sees to the welfare of their members. At the time of the study, the population of commercial intra-city drivers under the NURTW, RTEAN and Cooperative Union of Road Transport Workers were 88, 75 and 60 respectively. Overall, 300 drivers were employed by the Osun State Government in the Department of General Services, State Ministry of Works and Transport for driving official vehicles. Facilities providing Eye care services in Osogbo were Ladoke Akintola University of Technology (LAUTECH) Teaching Hospital - a tertiary health facility; State Specialist Hospital- a secondary health facility; and six private specialist hospitals namely Jolaolu Specialist Eye Hospital, JJ Specialist Eye Hospital, Grace Specialist Eye Hospital, Minaret Specialist Eye hospital and Onward Specialist Hospitals.

**Study design:** this was a comparative cross-sectional study design.

**Study population:** the study population was male commercial intra-city mini-buses' drivers and drivers employed by the Osun State Government working at the State secretariat in Osogbo metropolis. The commercial and public institution driving comprises mostly of males in Nigeria. We included commercial intra-city mini-buses drivers registered under Osogbo branch of the selected supervisory bodies and drivers employed by the Osun State Government working at the State secretariat, who have been driving for at least 1 year prior to the study. Commercial intra-city mini-buses drivers registered under Osogbo branch of the selected supervisory bodies and drivers employed by the Osun State Government working at the secretariat who were sick during the study period and those who declined participate in the study were excluded.

**Sampling:** the sample size was calculated using the formula for comparing two groups:

$$\text{n}=\frac{2{\left(\text{Zα+Zβ}\right)}^{2}*{\text{P}}_{0}(1-{\text{P}}_{0})}{{\text{d}}^{2}}$$

Where n is the minimum sample size, Zα critical ratio at significance level of 5%, Zβ Statistical power for one-sided test at 90%, P_0_ Average of the 2 prevalence in the 2 comparison groups and d difference between P_1_and P_2_. The minimum sample size was 112 for each group. However, 10% of the minimum sample size was added to make about 124 in order to adjust for non-response and improperly filled questionnaires. One hundred and twenty respondents were eventually selected for each group giving a total sample size of 240 respondents and response rate of 96.8%.

**Sampling technique:** multistage sampling technique was used in selecting the intra-city commercial drivers. Olorunda Local Government Area (LGA) was selected using simple random sampling by balloting from the three Local Governments in Osogbo metropolis. Two road transport unions (NURTW and RTEAN) of the mini-buses was selected using simple random sampling by balloting from Olorunda LGA. Drivers in the two road transport unions (NURTW and RTEAN) were line-listed, with 88 drivers registered in NURTW and 75 drivers in RTEAN. A proportionate to size allocation was used to select the number of drivers to be interviewed in both road transport unions (NURTW and RTEAN) which were 65 and 55 drivers respectively. Eligible respondents from each association were selected by systematic random sampling, making a total of 120 respondents among the commercial intra-city drivers. Drivers in the State secretariat (300 in number) were line-listed and 120 respondents were selected by systematic random sampling from the Government employed drivers.

**Data collection:** data was collected using a structured pre-tested questionnaire to obtain information on socio-demographic and economic characteristics, knowledge of drivers about visual dysfunction, attitude towards visual dysfunction and health seeking behavior for visual dysfunction and barriers for not seeking medical treatment. The questionnaire was translated from English to Yoruba which is the predominant language in the study area, for easy of understanding and uniform interpretation of the questions and back translated to English afterwards to ensure there was no ambiguity in translation. Ten trained medical students as research assistants in pairs, administered questionnaires in Yoruba language to study participants. The principal investigator supervised pairs of interviewers during data collection to ensure interviews were conducted appropriately. The interviews lasted an average of 10-15 minutes per respondent.

**Data processing and analysis:** the questionnaires were manually sorted out to check for errors and omissions at the end of data collection. Data was entered and analysed using Statistical Package for Social Sciences (SPSS) version 17. The questions on awareness about visual functions and attitude of the respondents concerning visual problems were scored. For questions whose responses were either yes or no (or correct and incorrect), a correct answer was scored 1 and a wrong answer was scored 0. For questions with three responses, (yes, no and not sure) the correct response was scored 2, the wrong response was scored 0, and not sure was scored 1. For the questions about attitude that had strongly agree, agree, indifferent, disagree and strongly disagree options, the responses were scored 5, 4 3, 2 and 1 in that order for a positive attitude and 1, 2, 3, 4 and 5 for a negative attitude. The maximum scores possible for awareness and attitude were 63 and 70, the mean scores calculated were 31.8 ± 7.9 and 20.3 ± 6.5 respectively. The respondents who scored below the mean were regarded as having poor awareness and negative attitude, while those who scored up to or above the mean were regarded as having good awareness and positive attitude. Health seeking behavior was adjudged good or bad based on respondents' awareness and management of visual dysfunction and where help was sought from for their visual dysfunction. Mean knowledge and attitude between both groups of drivers were compared across socioeconomic characteristics. Statistical analysis of difference between means was done by the use of two sample independent t-test to determine association between mean differences of knowledge, attitude and health seeking behavior for visual dysfunction. Level of significance was set with p-value less than 0.05 or 95% confidence interval.

**Ethical consideration:** Ethical clearance for the study (Protocol number: LTH/EC/2013/05/0143) was obtained from LAUTECH Teaching Hospital Ethical Review Committee. Permission to carry out the study was obtained from the Executive bodies of the RTEAN & NURTW Olorunda LGA and the Permanent Secretary, Department of General Services State secretariat Abeere. Reasons for the study were explained in detail to the respondents and participation was voluntary. Verbal consent was obtained from the respondents. All information gathered was kept confidential; participants were identified using only serial numbers. Data was stored securely in password-protected computer.

## Results

One hundred and twenty out of one hundred and twenty-four questionnaires were returned, properly filled and analyzed, giving a response rate of 96.8%. Forty-eight (40.0%) government drivers were at least 50 years of age (Mean age: 44.5years±8.5) and 58 (48.3%) had secondary education. Fifty-five (45.8%) commercial drivers were between the age of 30-39 years (Mean age: 38.5±8.6) and 63 (52.5%) had secondary education. At the least, two (1.7%) government drivers were between 20-29 years of age as compared to 14 (11.7%) commercial drivers. More government drivers 21 (17.5%) had tertiary education as compared to their commercial counterparts 4 (3.3%) (Table 1). Twelve (17.0%) government drivers have had eye test done as requirement for obtaining driver's license as compared to 8 (66.7%) commercial drivers. Government drivers 116 (96.7%) were more knowledgeable of all the visual defects that could constitute problems than their commercial counterparts 54 (45.0%). There was significant difference in the level of knowledge for both groups (p=0.000). The mean difference of educational status among the government and commercial drivers with regards to knowledge of visual dysfunction were found to be statistically significant (p=0.000). The mean age difference for government and commercial drivers across the age groups was also found to be statistically significant (p=0.000) (Table 2). Government drivers 108 (90.0%) had more positive attitude concerning visual problems compared to the commercial drivers 17 (14.2%). The difference in respondents' attitude concerning visual problems in both study groups were statistically significant (p=0.000). The mean difference of educational status among the government and commercial drivers with regards to attitude towards visual dysfunction were found to be statistically significant (p=0.000). The mean monthly income difference for government and commercial drivers across the age groups was also found to be statistically significant (p=0.000) (Table 3). Health seeking behavior of government and commercial drivers was demonstrated by conduct of routine eye examination and awareness of visual defects in them. More government drivers, 70 (58.3%) have had eye test done before than commercial drivers 20 (16.7%) (p<0.001). The main reason for the eye test was for official purposes among the government drivers. Other reasons were participation in free eye test and personal decision to have eye test done. Twenty-four (20.0%) government drivers were aware of their current visual problems before the conduct of the study while none of the commercial drivers were aware of visual defects in them (p<0.001). Of the 24 government drivers, 10 (47.6%) visited the hospital for treatment with eye examination done and recommended spectacles for correction of visual dysfunction. Busy workplace schedule (20.8%, n= 5) and lack of awareness of visual defects (12.5%, n = 3) were some barriers for not seeking medical treatment. The mean knowledge and attitude differences of the government and commercial drivers with regards to visual dysfunction as demonstrated by their health seeking behaviours were found to be statistically significant (p=0.000) (Table 4).

**Table 1:** Socio-demographics of government and commercial drivers in Osogbo, Osun State, Nigeria - 2015

Characteristics	Driver type		χ2	df	p-value
	Government N = 120 n (%)	Commercial N = 120 n (%)			
**Age (in years)**					
20-29	2 (1.7)	14 (11.7)	25.07	3	<0.001*
30-39	34 (28.3)	55 (45.8)			
40-49	36 (30.0)	30 (25.0)			
≥ 50	48 (40.0)	21 (17.5)			
Mean age	44.53 ± 8.51	38.52 ± 8.60			
**Educational level**					
Primary	41 (34.2)	53 (44.2)	13.30	2	0.001*
Secondary	58 (48.3)	63 (52.5)			
Tertiary	21 (17.5)	4 (3.3)			
**Religion**					
Christianity	61 (50.8)	31 (25.8)	15.86	1	<0.001*
Islam	59 (49.2)	89 (74.2)			
**Monthly income (in naira)**					
< 18,000	3 (2.5)	32 (26.7)	28.13	1	<0.001*
≥ 18,000	117 (97.5)	88 (73.3)			

* Statistically significant

**Table 2:** Knowledge of visual dysfunction of government and commercial drivers in Osogbo, Osun State, Nigeria - 2015

Characteristics	Driver type				p-value	95% CI
	Government		Commercial			
	N	Mean ± SD	N	Mean ± SD		
**Age (in years)**						
20-29	2	42.00±0.00	14	30.79±5.73	0.018	2.26-20.16
30-39	34	35.85±2.06	55	27.42±7.33	<0.001	5.87-10.99
40-49	36	36.03±3.33	30	29.07±8.23	<0.001	3.97-9.95
≥ 50	48	37.27±3.66	21	21.05±9.98	<0.001	12.95-19.49
**Educational level**						
Primary	41	35.80±3.43	53	28.25±8.00	<0.001	4.89-10.20
Secondary	58	36.72±3.30	63	25.97±8.85	<0.001	8.31-13.19
Tertiary	21	37.67±2.50	4	30.00±0.00	<0.001	5.04-10.30
**Religion**						
Christianity	61	37.07±3.22	31	26.71±8.60	<0.001	7.89-12.82
Islam	59	36.07±3.26	89	27.25±8.35	<0.001	6.56-11.08
**Monthly income (in naira)**						
< 18,000	3	42.00±0.00	32	25.69±9.64	0.01	4.83-27.79
≥ 18,000	117	36.44±3.19	88	27.63±7.88	<0.001	7.23-10.39

*Statistically significant

**Table 3:** Attitude towards visual dysfunction of government and commercial drivers in Osogbo, Osun State, Nigeria - 2015

Characteristics	Driver type				p-value	95% CI
	Government		Commercial			
	N	Mean ± SD	N	Mean ± SD		
**Age (in years)**						
20-29	2	28.00±0.00	14	18.07±4.39	0.01	3.07-16.79
30-39	34	23.82±1.93	55	16.73±6.31	<0.001	4.87-9.31
40-49	36	24.14±3.27	30	17.73±6.38	<0.001	3.98-8.84
≥ 50	48	25.00±2.81	21	11.52±7.12	<0.001	11.11-15.85
**Educational level**						
Primary	41	23.71±2.69	53	17.08±5.47	<0.001	4.78-8.48
Secondary	58	24.53±2.80	63	15.33±7.52	<0.001	7.12-11.28
Tertiary	21	25.71±2.55	4	19.00±1.15	<0.001	3.99-9.43
**Religion**						
Christianity	61	37.07±3.22	31	26.71±8.60	<0.001	7.89-12.82
Islam	59	36.07±3.26	89	27.25±8.35	<0.001	6.56-11.08
**Monthly income (in naira)**						
< 18,000	3	28.00±0.00	32	15.63±6.57	0.002	4.55-20.19
≥ 18,000	117	24.37±2.77	88	16.44±6.63	<0.001	6.59-9.27

*Statistically significant

**Table 4:** Health seeking behaviour for visual dysfunction of government and commercial drivers in Osogbo, Osun State, Nigeria - 2015

Characteristics	Driver type				p-value	95% CI
	Government		Commercial			
	N	Mean ± SD	N	Mean ± SD		
**Age (in years)**						
20-29	2	4.00±0.00	14	4.43±0.52	0.28	-1.24-0.38
30-39	34	4.41±0.50	55	4.00±0.00	<0.001	0.28-0.54
40-49	36	4.64±0.49	30	4.13±0.36	<0.001	0.29-0.73
≥ 50	48	4.63±0.49	21	4.48±0.51	0.25	-0.11-0.41
**Educational level**						
Primary	41	4.44±0.50	53	4.11±0.32	0.0002	0.16-0.50
Secondary	58	4.62±0.49	63	4.22±0.42	<0.001	0.24-0.56
Tertiary	21	4.62±0.50	4	4.00±0.00	0.02	0.09-1.15
**Religion**						
Christianity	61	4.66±0.48	31	4.19±0.40	<0.001	0.27-0.67
Islam	59	4.46±0.50	89	4.16±0.37	<0.001	0.16-0.44
**Monthly income (in naira)**						
< 18,000	3	4.00±0.00	32	4.19±0.40	0.05	-1.14-0.00
≥ 18,000	117	4.57±0.50	88	4.16±0.37	<0.001	0.29-0.53
**Knowledge of visual dysfunction**						
Good	116	4.56±0.50	54	4.22±0.42	<0.001	0.19-0.49
Poor	4	4.50±0.58	66	4.12±0.33	0.04	0.03-0.73
**Attitude towards visual dysfunction**						
Positive	108	4.53±0.50	17	4.12±0.33	0.001	0.16-0.66
Negative	12	4.83±0.39	103	4.17±0.38	<0.001	0.43-0.89

*Statistically significant

## Discussion

The mean age of 39 years in the commercial drivers is similar to what was reported [15] in a previous study and 45 years in government drivers which was lower than another study conducted on drivers in public institution in Ibadan [18]. The older age observed among government drivers could be because they are required to be educated at the time of recruitment into civil service. The mean ages in both study groups is however, in keeping with an active work force that the respondents belong to as commercial and public institution driving is a demanding occupation. Safe driving is a function of driver's age. With increasing age, there is a decline in sensory cognitive function. It has been reported that older drivers have more accidents per mile than their younger counterparts [19]. The level of education of respondents showed that less than a quarter of government drivers had post-secondary education whereas less than a tenth of commercial drivers had post-secondary education, similar finding was found in studies conducted in south western part of Nigeria [7, 13]. Government drivers were significantly better educated than commercial vehicle drivers and this may explain why they also had more periodic eye examination than commercial vehicle drivers since their level of health awareness may be higher. Another reason for better educational attainment and more frequent eye check may be because government drivers are often of higher socioeconomic status. In relation to income, more of the government drivers earned above 18,000 naira than the commercial drivers. This presupposes that the government drivers have more financial capability to attend to their health matters as compared to their commercial counterparts. The mean differences of age, educational status and monthly income among the government and commercial drivers showed that government-employed drivers were more knowledgeable on visual dysfunctions as compared to their commercial counterparts. The knowledge gap on visual functions and awareness of dysfunctions between government and commercial drivers could explain the conduct of survey with emphasis on the commercial drivers [6, 7, 13-15].

Also, this difference was statistically significant mostly because of improved knowledge of government-employed drivers generally about visual functions. The high level of knowledge among the government-employed drivers could be as a result of the fact that majority of the government drivers have heard of these eye problems through sensitization and awareness programs concerning the eye conducted by experts, organized by the state government and also by non-governmental organizations dealing with matters relating to the eye of which their commercial counterpart was not privy to. Government drivers had a better positive attitude towards visual dysfunction as compared to their commercial counterparts, awareness of visual defects and willingness to be treated was found to be more common with findings similar to a study in Ibadan, south-west Nigeria [8]. The mean differences of age, educational status and monthly income among the government and commercial drivers showed that government-employed drivers were of a better positive attitude towards visual dysfunctions as compared to their commercial counterparts. Concerning respondents' health seeking behavior for visual dysfunction, more than half of government drivers as compared to about a fifth of commercial drivers have had eye examination done before the conduct of the study. This was however, quite low and surprisingly in agreement with some other studies [6, 7, 13, 20-26] due to the fact that majority of drivers don't even do eye test before obtaining license. About half of the government drivers that had previous eye examination were attributed to official reasons, possibly as part of pre-employment medical examination for newly employed workers. This was commendable and should continue to be observed. Also, two out of ten government drivers were aware of visual problems in them, whereas none of the commercial drivers were aware of visual problems in them. This finding was also in agreement with studies conducted in the southwestern part of Nigeria [13, 18, 27, 28]. We found that government drivers had good health care seeking behaviour as compared to their commercial counterparts. This was reflected in their knowledge and attitude towards visual dysfunctions which later played out in their health seeking behavior as it relates to their visual function. Busy schedule at workplace and ignorance of visual status were barriers that prevented some of the government drivers from going to the hospital. This finding was similar to studies conducted in Plateau State [14] and Ghana [15]. The mobile nature of the commercial and government drivers was put into consideration and data collection was done during the road transport workers meetings for commercial drivers and departmental meeting of government drivers.

## Conclusion

Our study revealed that knowledge and attitude towards visual dysfunction was significantly higher among the government drivers than their commercial counterparts. Also, some of the government drivers were aware of their visual problems; however, none of the commercial drivers was aware of any visual problem in them. Government drivers had good health seeking behavior for visual dysfunction compared to their commercial counterparts. We recommended routine eye medical check-up for early detection of visual dysfunction in vehicle motor drivers.

### What is known about this topic

- Knowledge and attitude of motor vehicle drivers regarding visual function.

### What this study adds

- Health seeking behavior of motor vehicle drivers regarding visual dysfunction.

## Competing interests

The authors declare no competing interests.
